# Advancing three-dimensional tendon imaging using laboratory X-ray phase contrast techniques and refined sample preparation

**DOI:** 10.1038/s41598-026-57551-w

**Published:** 2026-06-13

**Authors:** Charlotte J. Maughan Jones, Jayesh Dudhia, Alberto Astolfo, Alessandro Olivo, Charlotte Hagen

**Affiliations:** 1 Department of Medical Physics and Biomedical Engineering, University College London, London, UK; 2 Centre for Engineering Education, University College London, London, UK; 3 Department of Clinical Sciences and Services, Royal Veterinary College, Hertfordshire, UK

**Keywords:** X-ray phase contrast, Tendon, Edge illumination, Biological techniques, Biotechnology, Engineering, Medical research

## Abstract

Tendinopathy is of great socio-economic importance, with high rates of prevalance in both athletic and non-athletic populations. Despite this, there remains limited understanding of the three-dimensional macro and microscopic anatomy and its significance in health, clinical and sub-clinical disease due to difficulties in gaining three-dimensional images of tissue volumes. Although histology is considered the gold standard for pre-clinical tendon imaging, the tissue is notoriously difficult to process and section, leading to a high incidence of artefacts. X-ray phase contrast imaging (XPCi) is becoming increasingly important with regards to three-dimensional imaging of biological tissues, and has shown promise in tendon imaging with synchrotron radiation, however laboratory based imaging and associated sample preparation protocols have yet to be validated. samples in this work, equine superficial digital flexor tendons were prepared using various combinations of PBS, 10% neutral buffered formalin, 70% and 100% ethanol and imaged using a laboratory based edge illumination XPCi system in a custom made 3D printed container. Consistent with other findings for tissue, contrast for tendon tissue was found to be maximised when dehydrated in ethanol, while the fixation medium has no notable affect on contrast.

**Supplementary Information:**

The online version contains supplementary material available at https://doi.org/10.1038/s41598-026-57551-w.

## Introduction

Tendon disease involving degeneration of the internal structure and change in biomechanical properties of the tendon microstructure (tendinopathy) is of substantial socio-economic importance. It is highly prevalant amongst athletes, and specific working populations, with reported rates of up to 23.3%^1^ and 41%^2^ respectively. Diagnosis remains largely based on clinical signs; yet imaging techniques such as MRI and ultrasound often leads to incidental findings of the disease in asymptomatic individuals, suggesting substantial underestimation of true disease prevelance^2–4^. Although the full economic burden of tendinopathy remains underexplored, musculoskeletal conditions account for over £5 billion in National Health Service (NHS) expenditure annually in the UK^5^. Additional costs arise from lost productivity, with work absence due to lateral epicondylitis (tennis elbow) alone estimated to have cost £27 million in 2012^2^. Despite the symptomatic and asymptomatic prevelance, and socio-economic impact, the development of effective treatment and management strategies is hindered by an incomplete understanding of the pathophysiology of diease and how this is linked to the three-dimensional macro and microscopic structure of normal and diseased tendon tissue^6^.

Horses represent a well-established spontaneous model of tendinopathy^7^ allowing the study of disease mechanisms. Not only is this beneficial for humans, but also for horses themselves due to the high prevalence of tendon injury within equine athletic populations, reported to be as high as 24%^8^. As such, using the horse model aligns with the One Health principle^9^ promoted by the World Health Organisation, which advocates for integrated research strategies to improve the understanding and treatment of conditions common to both humans and animals^5,6^.

Histology remains the current gold standard for investigating the internal three dimensional structure of healthy and diseased tendon tissues, however tendon’s dense, and highly organised hierarchical architecture, composed primarily of highly aligned collagen fibers arranged in a multi-scale architecture – from fibrils to fascicles – is particularly succeptible to histological processing artefacts. Standard histological processing which involves chemical dehydration, xylene clearance, parrafin embedding and microtomy often results in tearing, fraying and hardening in tendon tissues, regardless of the plane in which it is cut^10,11^. This can interfere with interpretation of results, preventing effective correlation with clinical presentations. These challenges motivate the development of techniques that can be applied to image tendon microstructure in 3D. Furthermore and in general, histology remains essentially a 2D technique; although efforts to produce three dimensional data sets from histological slices have been made in a variety of tissues, however 3D reconstruction remains challenging due to preparation induced distortions to the tissue, hampering accurate registration of features between slices^12^. Digital reslicing of reconstructed three dimensional histological datasets in different planes can also lead to a lower out-of-plane resolution^13^ which is physically limited by slice properties such as thickness, or slice frequency^14^. To overcome the challenges of tendon histology, optical based imaging methods have been utilised to understand the three-dimensional structure, however they are limited by their small field of view, limited optical penetration into the highly scattering tendon, or by extensive tissue processing known as ‘optical clearance’^15–17^.

X-ray phase-contrast imaging (XPCi) represents a potential alternative, offering a high-resolution, non-destructive imaging capability for tendon tissues that would facilitate greater understanding of its three-dimensional anatomy in both health and disease. It has already shown promise for preclinical imaging due to its ability to provide increased contrast in biological tissues compared to traditional x-ray imaging, without the need for exogenous contrast media or destructive sample processing methods as in histology^18–20^. Standard x-ray based imaging methods rely on the difference in absorption properties of materials to produce contrast within an image. XPCi however also utilises the refractive properties of tissues, which can be up to three order of magnitude greater than the corresponding absorption properties, resulting in greater intrinsic contrast^11,12^. In terms of application to tendon tissue, XPCi has already demonstrated promise in studying collagen fibres in fresh-frozen as well as formalin fixed, cell macerated and decalcified murine tendon^21–24^. However, due to differences between mouse and human anatomy, mouse models have limited translation potential^9^. Horse tendon, which presents a more suitable model for understanding human disease, and has not yet been studied with XPCi. Furthermore, in the murine studies synchrotron-based XPCi had been used, which is somewhat limiting due to the restrictive access to synchrotron facilities. This work explores the application of the laboratory based, incoherent and polychromatic edge-illumination XPCi (EI-XPCi) to horse tendon.

Although it would be ideal to image all tissue fresh (that is, without any processing applied), which some have sucessfully done^23–27^, long acquisition times at room temperature in laboratory based settings render fresh tissue liable to a combination of autolysis and uncontrolled dehydration, which can lead to cellular changes, decomposition and alteration in sample dimension and morphology. This is of particular importance to tendon tissue, whose three-dimensional structure is intrinsically linked to its function. Fresh tissues can also pose safety concerns for personel due to potential infectious or (when using animal models of disease) zoonotic disease transmission, which may be a health and safety consideration within imaging laboratories. Overarchingly, fresh tissue tends to be difficult to image, especially with techniques that tend to incur lenghty scan times, such as XPCi. Sample processing is therefore required to maintain the biological anatomy, whilst making ‘risky’ tissue safer. As is shown within this study, sample processing can also lead to enhanced image contrast.

A significant barrier to the widespread adoption of XPCi for tendon speficically, but also for biological tissues in general, as a viable alternative or complement to established histological processes is the lack of consensus on the most effective sample preparation technique. There are varied approaches reported in the literature, ranging from formalin fixation^28^, paraffin embedding^29^, aldehyde fixation and osmium impregnantion^30^, chemical dehydration^31^ and critical point drying^32^. Many of the reported approaches still suffer from issues such as bubble formation^33^ or long sample preparation times^31^. Reported approaches have focused on brain, heart, lung, kidney, spleen^31^, spinal cord^30^ and other tissues, however the translation of the findings to low cellularity, dense tissues, specifically tendon, remains underexplored.

The purpose of this paper is three-fold. By applying a variety of simple and common sample preparation techniques which present stark differences in contrast and feature identification, we demonstrate the need for agreeing upon a reproducible sample preparation pipeline for tendon imaging in XPCi. Furthermore, and consistent with previous findings for other tissue types^18,31^, our results demonstrate that the application of a chemical dehydration agent (ethanol) improves contrast for tendon tissue relative to the fresh tissue state, and thus propose its use as a recommended step prior to the application of edge illumination XPCi in future tendon studies. Finally, this proof of concept study presents EI-XPCi as a potential alternative to the histopathological examination of tendon tissues, which, as an inherently 3D technique, may be used to facilitate greater understanding of the architecture of this challenging-to-slice tissue in health and disease.

## Methods

### Tissues

Horses are a spontaneous and established model of human tendon disease. The superficial digital flexor tendon (SDFT) in horses is the functional equivalent of the Achilles tendon in humans: as elastic energy storing tendons, both share remarkable ageing phenotypes^34^ and prevelance of injury (tendinopathy) as a result of athletic activity^35^. SDFT were therefore chosen for this study in line with the “One Health” principle, as promoted by the World Health Oragisation, which aims to advance the understanding of, and provide mutually beneficial health benefits for, both humans and animals.

Normal (healthy) SDFT tissue was excised within 24 h of post-mortem from Thoroughbred-type horses that had been euthanised at an abattoir (Wiltshire, UK) for reasons unrelated to this study. Dissected SDFT tissue from the mid-metacarpal was flash frozen in n-hexane at − 80 °C and stored at − 80 °C at the Royal Veterinary College. The tissue was subsequently defrosted at room temperature and cut into 3 equal lengths (approximately 2 cm each axially) ready for processing.

### Tissue processing

**Fig. 1:**
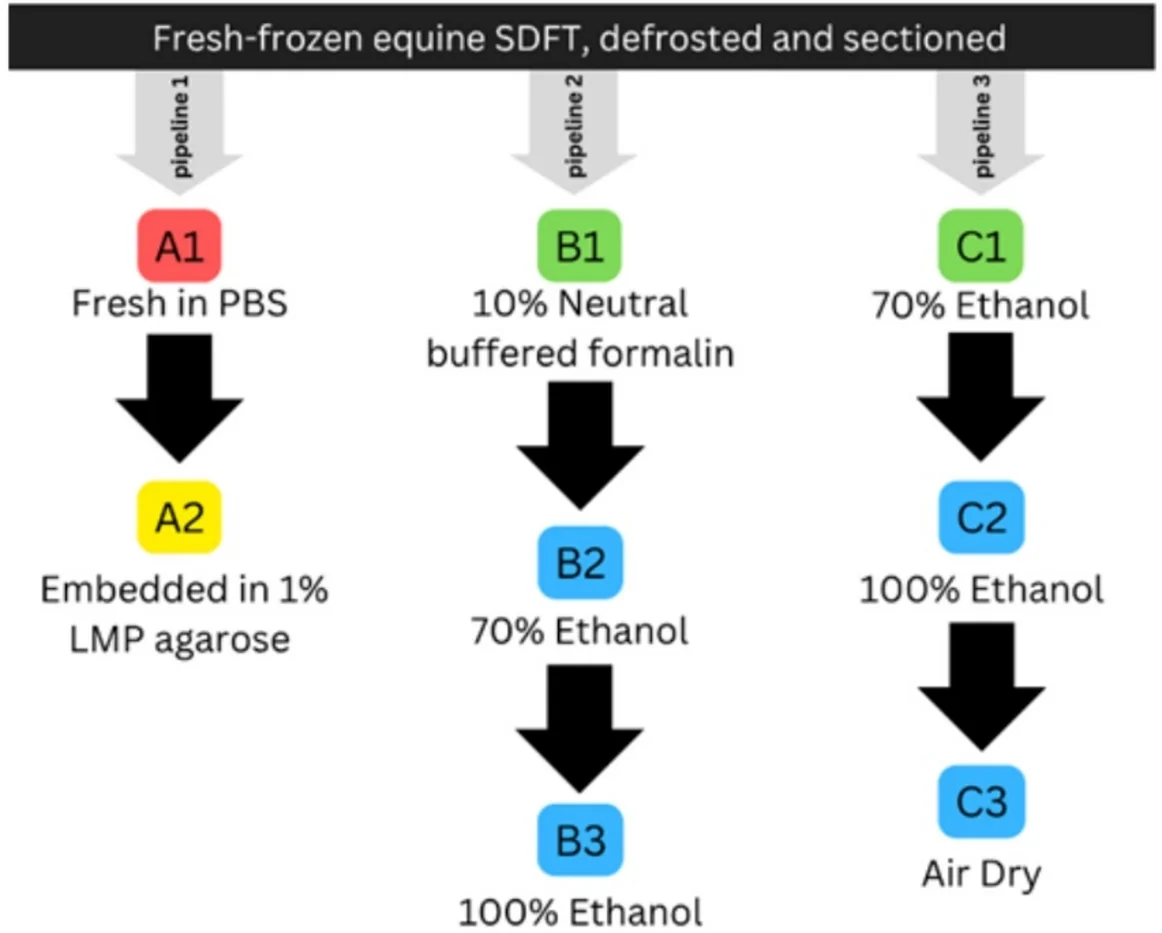
Flow diagram demonstrating the tissue processing pipelines for the three equine tendon samples. A total of eight preparation techniques, labelled A1–C3, were implemented. The three pipelines are composed of one of more of the following stages: storage (red), fixation (green), dehydration (blue) and embedding (yellow).

Each of the 3 tissue lengths (referred to as samples A, B, C in the following) was processed according to one of three pipelines shown in Fig. 1, resulting in 8 distinct preparation techniques (A1-C3).

#### Storage

All tissues were stored at − 80 °C immediately after *ex vivo* dissection as previously described. Once defrosted, they were stored in phosphate buffered saline (PBS) to ensure mechanical and chemical stability of the tissues whilst in short term storage, for a maximum of 24 h prior to further processing.

#### Fixation

Tissues were fixed in one of two ways: aldehyde fixation (pipeline 2) via commercially available pre-filled containers of 10% neutral buffered formalin (NBF), or alcohol fixation (pipeline 3) via 70% ethanol (70% EtOH) which was made by diluting 100% ethanol (100% EtOH) with deionised water. In each case, the sample was submerged in approximately 60 ml of liquid for a minimum of 48 h to ensure complete fixation. Tissues were stored within their fixation liquid until imaging or further processing.

#### Dehydration

Initial partial dehydration of tissue was achieved via submersion in 70% ethanol. Complete dehydration was achieved via manual immersion in 200 ml of a gradually escalating ethanol series (80%, 80%, 90%, 100%, 100%, 100%) for 45 min per step, in line with previously described protocols^18,36^. Tissues were stored in either 70% or 100% ethanol until imaging or further processing.

Air dried samples were left at room temperature for 24 h in an open container to allow evaporation of residual ethanol in a completely uncontrolled process.

#### Embedding

A 1% low melting point agarose (LMPA) was prepared by boiling 100 ml PBS with 1 g of LMPA powder for one minute on a hot plate until the powder had fully disolved. LMPA was used due to the lower temperature of gelation, meaning the hot solution could be cooled to approximately 40 °C to prevent heat damage to the tissue prior to solidification. Once cooled, the solution was carefully pipetted around the sample in the container. Care was taken not to introduce any bubbles. The embedded sample was then refridgerated at 4 °C until the agarose had set and the sample was stored in this way until imaging.

Although formalin fixation followed by paraffin embedding is widely considered gold standard for clinical hisopathology, this was not considered within this study as the emphasis was on simple preparation techniques with minimal processing steps, and the known challenges of slicing tendon tissue.

### X-ray imaging

#### Sample mounting

A simple reproducible and watertight sample container and lid were designed using open access online CAD software Autodesk Tinkercad (https://www.tinkercad.com/) and 3D printed using thermoplastic polylactic acid (PLA) via the Ultimaker S5 3D printer and associated Ultimaker Cura software (version 5.0). Design and detailed printing parameters have been made available via an online repository by the authors^37^. The tube was printed using the ‘spiralise’ setting, creating container walls of approximately 0.4 mm thickness - the resolution limit of the 3D printing system. PLA was chosen as it is chemically compatible with ethanol, formalin and PBS, and minimally absorbs the incident x-ray beam. The container was designed with a 2 cm base height as this positioned the sample precisely within the x-ray beam without adjustment to any system hardware to ensure reproducibility in positioning between samples.

**Fig. 2:**
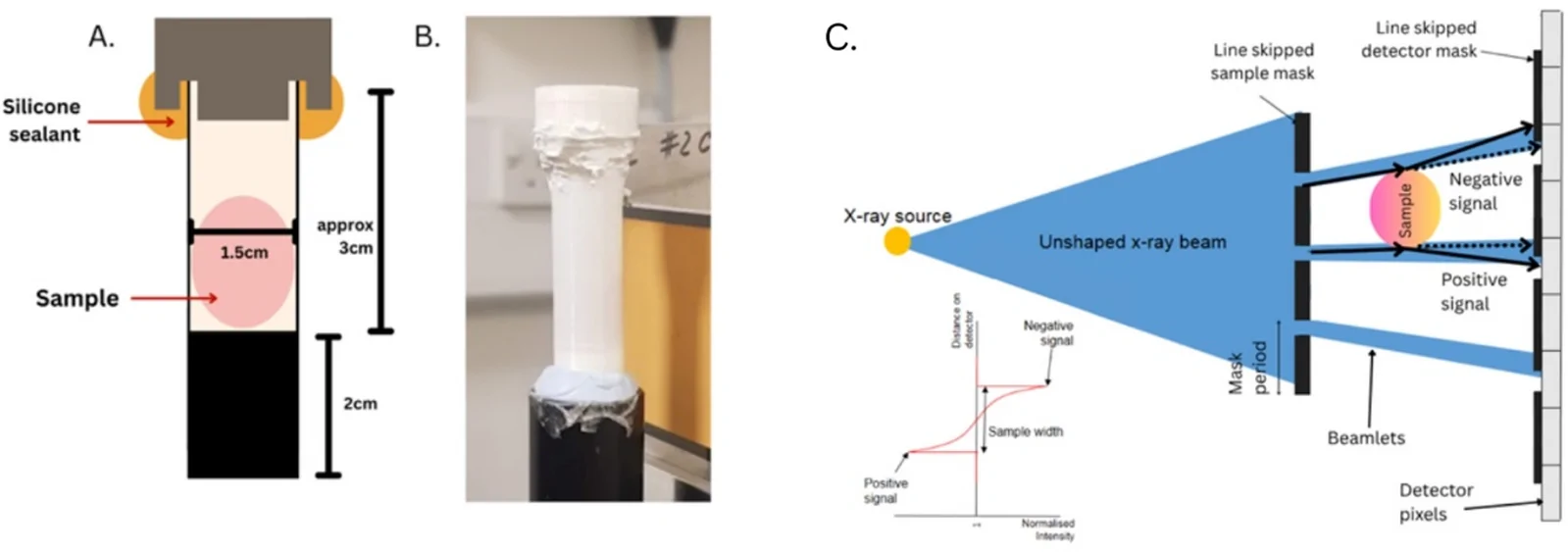
(**A**) Schematic cross section of 3D printed sample container demonstrating the key dimensions. (**B**) Photograph of fully sealed and mounted sample container within the EI-XPCI system and (**C**) of EI-XPCi line skipped system, demonstrating the process of signal formation.

Once the sample (longitudinally) and any dehydrating or fixation liquid were placed within the container, the lid was sealed shut with commercially available bathroom silicone sealant for added protection against accidental spills, and was then mounted on the sample stage ready for imaging (Fig. 2).

#### X-ray imaging system

Samples were imaged using a custom Edge illumination x-ray phase contrast imaging (EI-XPCi) system at UCL consisting of a rotating molybdenum anode source (MicroMax-007HF, Rigaku, Japan) run at 40kVp and 30 mA, emitting largely incoherent radiation with a polychromatic spectrum with a mean energy of approximately 18 keV. A schematic of the system can be seen in Fig. 2.

The source to detector and source to sample distances were set at 86 cm and 70 cm, respectively, with the detector mask held approximately 1.5 cm in front of the detector. The detector was a CMOS flat panel sensor (C9732DK Hamamatsu, Japan) with a pixel size of 50 μm. Detection of refraction caused by the sample is enabled by two ‘masks’ placed between the source and sample (sample mask) and the sample and detector (detector mask). The masks were manufactured by electroporating 100 microns of gold onto a graphite substrate leaving 10 micron transmitting apertures and a 79µm period in the sample mask, with the detector mask a magnified version based on the system geometry and divergence of the beam. The detector mask is aligned such that its apertures coincide with pixel centres, while the sample mask is aligned with a slight offset, such that the beamlets it creates fall partially onto the exposed pixel areas and partially onto the absorbing septa of the detector mask. Refraction then causes a deflection of the beamlets either onto the absorbing septa of the mask creating a negative signal, or onto the exposed pixel areas causing a positive signal (Fig. 2C). Note that, with the mask parameters given above, the detector mask fully covers every other column of detector pixels which would thus not be exposed to photons; this configuration, known as “line-skipping”, reduces the adverse effects of pixel cross talk^38^.

#### CT image acquisition parameters

Samples were rotated within the system allowing for images to be taken at every 0.2° over the full 360° angular range. In EI XPCi, the spatial resolution is decoupled from the source blurring and detector pixel size and is instead defined by the width of the apertures in the sample mask^39^. To leverage this property, samples were moved in 8 sub pixel steps with a 1.2s exposure image taken at each step - a process known as ‘dithering’^40^. Dithering was applied at each rotation angle. Flat and dark field images were taken before and after every 200 projections (flat field only) during the scan to facilitate gain and offset correction.

#### Image processing

Images were dark and flat field corrected, and the individual frames acquired during dithering combined into up sampled projections. These were then processed according to the so-called single image retrieval method developed for EI-XPCI^41^. Subsequently, the retrieved projections were reconstructed into axial CT slices via filtered back projection.

## Results

### Sample A

**Fig. 3:**
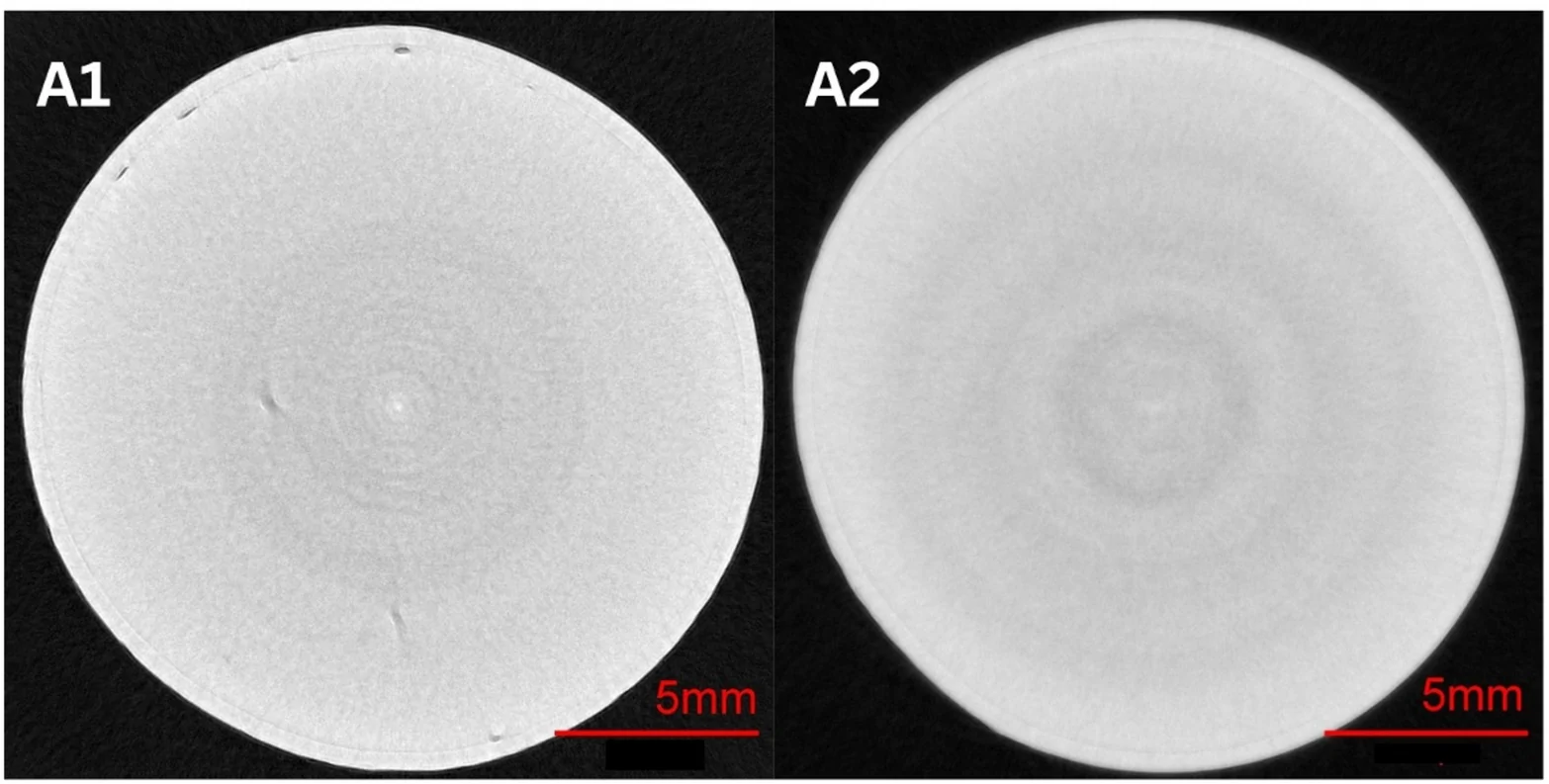
Axial CT slices of a sample container containing A1. Fresh SDFT tendon submerged in phosphate buffered saline (PBS), or A2. Fresh SDFT embedded in Low melting point agarose (LMPA).

Figure 3 shows CT slices for sample A, prepared according to the protocols A1 (left) and A2 (right). The images demonstrate the absence of contrast between the sample container, LMPA or PBS and the SDFT within it. It is practically impossible to discern the location of the tendon within the sample container, and no internal structural details can be visualised.

### Sample B

**Fig. 4:**
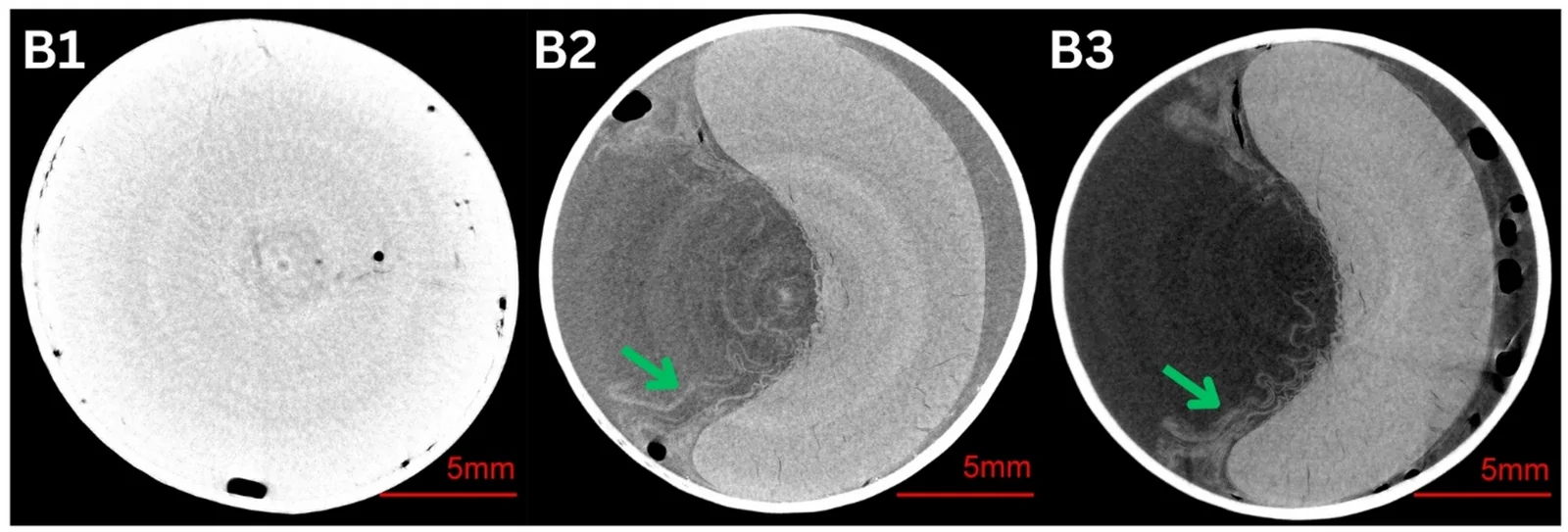
Axial CT slices of a sample container containing B1: Formalin fixed SDFT submerged in formalin, B2: Formalin fixed, 70% ethanol dehydrated SDFT submerged in 70% ethanol and B3: formalin fixed, dehydrated to 100% ethanol SDFT, submerged in 100% ethanol. Scale bars = 5 mm. Green arrow denotes loose connective tissue.

Figure 4 shows CT slices for sample B, prepared according to the protocols B1 (left), B2 (middle) and B3 (right). Fixing, and submerging fresh SDFT in formalin produces no contrast between the sample, the container and the formalin, again, leaving only traces of the tendon being visible within the container. Dehydrating the tissue in either 70% or 100% ethanol increases the contrast and allows some tissue detail to be observed such as the loose connective tissue, as seen in the close-up images shown in Fig. 6.

### Sample C

**Fig. 5:**
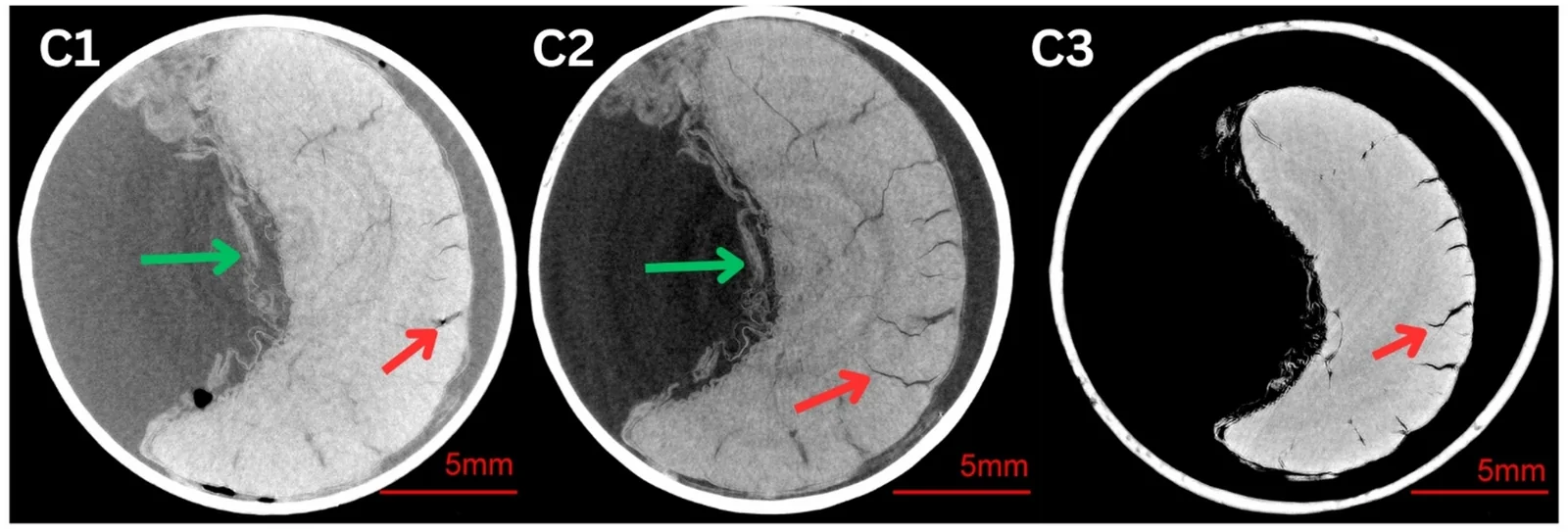
Axial CT slices of a sample container containing C1: 70% ethanol fixed SDFT, submerged in 70% ethanol, C2: 70% ethanol fixed, 100% ethanol dehydrated SDFT submerged in 100% ethanol and C3: air dried SDFT after previous processing. Scale bars = 5 mm. Green and red arrows depict loose connective tissue and cracks respectively.

**Fig. 6:**
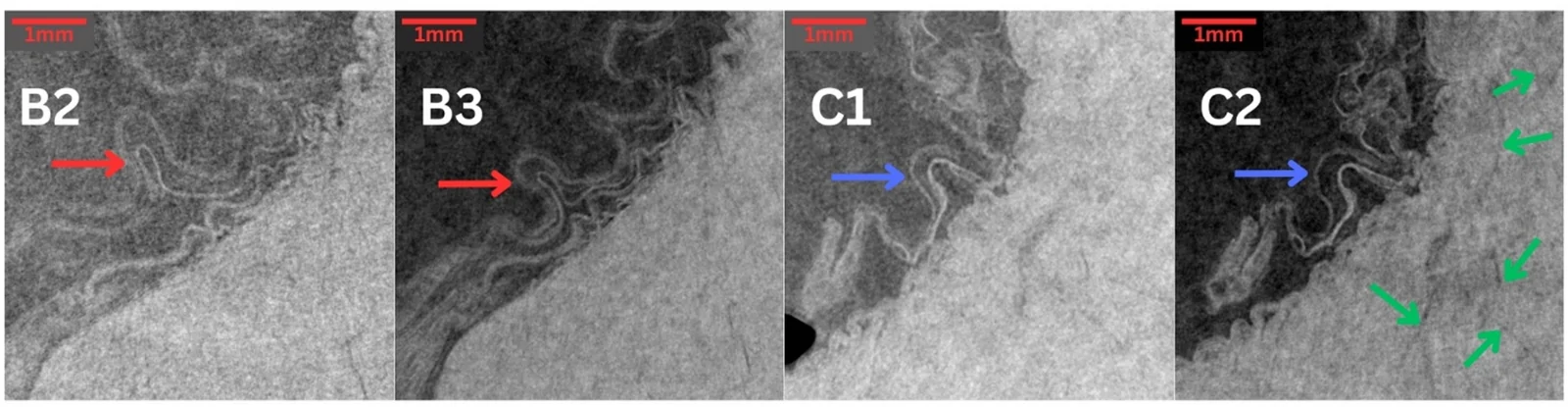
Close up detail of loose connective tissue in the samples prepared according to protocols B2, B3, C1 and C2. The red arrow indicated the same connective tissue feature in B2 and B3 and the blue arrow indicates a similar feature in C1 and C2. The green arrows denote internal structural details (fascicle boundaries), most visible in C2. Scale bar = 1 mm.

Figure 5 shows CT slices for sample C, prepared according to the protocols C1 (left), C2 (middle) and C3 (right). These show that fixing and submerging SDFT in 70% ethanol with or without dehydration in 100% ethanol produces contrast that facilitates identification of structures such as loose connective tissue and dehydration to 100% facilitates the faint visualisation of internal structural details (likely fascicle boundaries) as seen in the close-up images in Fig. 6. Fascicles are bundles of collagen fibres and are the largest subunit of tendons. Further uncontrolled air-drying causes significant shrinkage of the tendon (notable from the reduced size of the sample inside the container), which continued during the image acquisition resulting in blurring due to motion, and could be an indication that full dehydration had not yet occurred at the point of imaging. Although full dehydration was desired, due to the visible negative effects on the tissue (shrinkage and cracking) seen in Fig. 5 (C3), no further attempts to achieve full dehydration were pursued. To note, some features consistent with tissue cracking, possibly due to excessive or sudden dehydration, are also apparent in C1 and C2.

### Contrast to noise ratio (CNR)

CNR values were extracted from the images to facilitate a quantitative comparison. However, due to the lack of contrast in the images of samples prepared according to the protocols A1, A2 and B1 and the significant shrinkage in the C3 protocol, CNR was only considered for the images of the remaining 4 preparation techniques (B2, B3, C1, C2), as these remain the only viable techniques moving forward. CNR was calculated as per Eq. (1):

1 $$\:CNR=\:\frac{{\mu\:}_{T}-{\mu\:}_{B}}{{\sigma\:}_{B}}$$

**Fig. 7:**
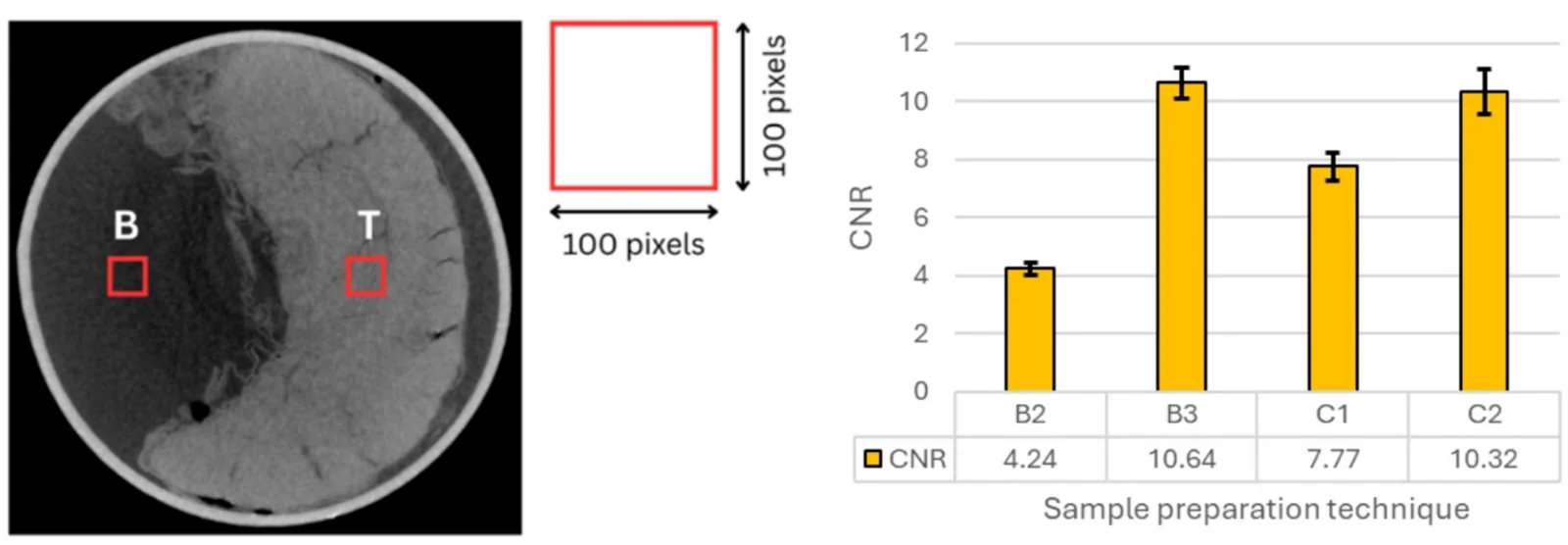
CNR in the images obtained from 4 of the sample preparation techniques. Error bars show +/- one standard deviation of CNR values calculated across the 100 slices.

Here, µ_T_ and µ_B_ denote the mean grey values in a tissue and background region and σ_B_ is the standard deviation in the background region, as depicted in Fig. 7. The location of the regions of interest (T and B) was checked and adjusted for each sample, to ensure that they were taken in an area without any visible artefacts, and with consistent contrast. Measurements were extracted across 100 CT slices for each sample. CNR results are displayed on the left in Fig. 7; each bar shows the average CNR over the 100 slices, with error bars corresponding to one standard deviation to illustrate interslice variations.

Figure 7 shows that both samples dehydrated to 100% (B3, C2) ethanol display greater CNR than those only dehydrated to 70% (B2 and C1), with sample B2 performing worst among the four considered preparation methods.

## Discussion

Ideally, imaging of biological tissues (including tendon) using XPCi should be performed on fresh *ex vivo* tissues to preserve native structure and clinical relevance. However, imaging fresh tissues pose challenges, including poor image contrast, susceptibility to autolysis, dehydration, and shrinkage during long scans in non-refrigerated systems. As shown above, these issues can be mitigated through carefully selected tissue processing techniques.

Results show that, in line with previous findings for other tissue types, image contrast for tendon tissue in XPCi is highly affected by the sample processing method, especially the presence, lack of and degree of dehydration. Samples that were not subject to any ethanol dehydration consistently demonstrated poor contrast regardless of the liquid/gel they were submerged/embedded in. This was found for both fresh and formalin fixed tissues. CNR can provide an insight into relative contrast differences given that all images are affected by the same degree of noise. Thus, contrast was found to be highest when tissues were dehydrated to 100% ethanol regardless of the fixation method. Processing tissues in 100% ethanol results in complete dehydration, whereas 70% ethanol leaves residual water within the tissue.

To explain these results, the principles behind contrast governance in XPCi can be examined. XPCi generates contrast through both attenuation and refraction, which are described by the complex index of refraction: = 1 − + , with the real (δ) and imaginary (β) parts relating to the attenuation and refraction (phase shifting) properties of a material, respectively. Tendon consists of approximately 70% water by mass with the remaining 30% largely collagen^42^; thus, tissue composition is notably altered by dehydration, which removes water from the cellular environment.

**Fig. 8:**
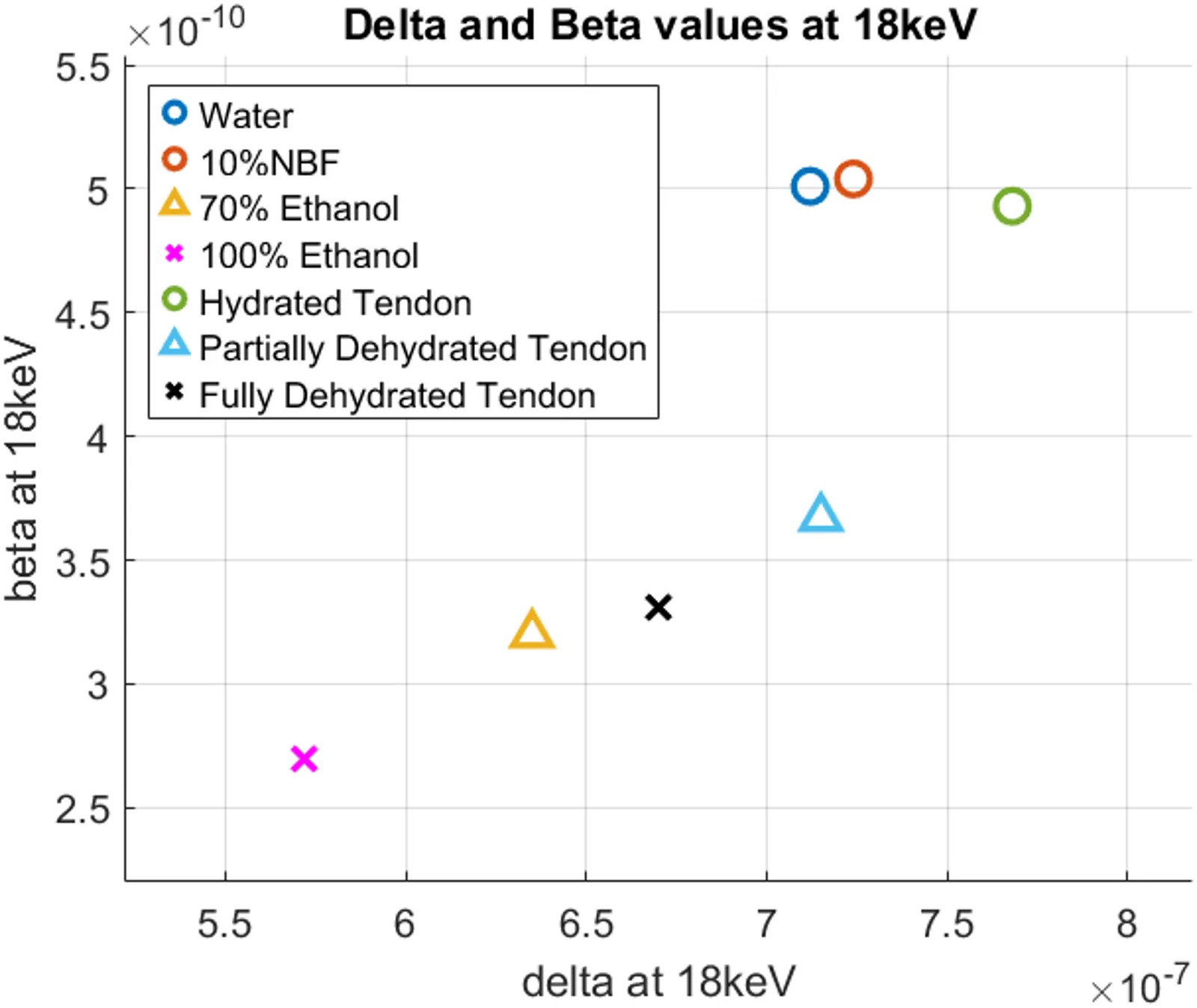
Calculated delta and beta values for tendon tissue and associated liquids.

To evaluate the impact of tendon tissue dehydration on XPCi, δ and β were calculated for tissue and the relevant substances used in the considered preparation techniques at 18 keV. The δ and β values of water, formalin and ethanol were obtained via an open-access X-ray refractive index calculator^43^, informed by density values and chemical compositions obtained from commercial datasheets^44,45^ and biomedical databases^46^. 10% Neutral Buffered Formalin (NBF) was modelled as 96% water and 4% formaldehyde and δ and β values were accordingly calculated as weighted averages, while Low Melting Point (LMP) agarose and Phosphate Buffered Saline (PBS) were treated as water-equivalent due to their high-water content. Collagen, the main constitute of tissue alongside water, is a structurally complex protein that lacks a definitive chemical formula; its δ and β values were thus approximated from calculated δ and β values for the amino acid residues present in equine collagen type I^47^. Density values for the amino acids were sourced from PubChem^46^, with approximations made using commercial datasheets where reference values were not available. To model progressive dehydration, three distinct hydration states of tissue were considered. The hydrated state consisted of a 7:3 ratio of water to collagen. Partial dehydration was simulated using a 7:3 ratio of 70% ethanol to collagen, while full dehydration was represented by a 7:3 ratio of 100% ethanol to collagen. Again, δ and β values were accordingly calculated as weighted averages. A full list of chemical formulas and density values used in the calculations is provided in the Supplementary Information, while δ and β values are plotted in Fig. 8.

Figure 8 shows that the difference between δ and β between 100% ethanol and fully dehydrated tissues is between 1.7 and 2.3 and 5.5 to 7.7 times greater respectively than the differences between hydrated tendon and its two submersion liquids (10% NBF and water). Partial dehydration shows an intermediate difference. This explains the higher contrast for samples dehydrated in 100% ethanol compared to those dehydrated in 70% ethanol, and also why fully hydrated tissues consistently produced little to no contrast when submerged in any aqueous liquid or material. This confirms that intrinsic tissue properties are not, on their own, capable of producing demonstrable and measureable contrast for fibrous, low cellularity tendon tissue within EI-XPCi techniques, and engineering of the intrinsic refractive index mismatch is required to observe contrast.

Although modification of δ through sample dehydration increases contrast, it is important to note that biological tissue, and in particular tendon tissue, can become hard and brittle when dehydrated. In fact, the lack of comparative histology slices in this study is due to this issue: even after one week of rehydration in saline solution followed by decalcification and paraffin embedding, samples remained too hard to slice via a microtime. This is a limitation of the approach that may need to be considered in applications where downstream histology is required alongside XPCi images.

Our results further show that the method of fixation (NBF or 70% ethanol) has negligible impact on the resulting contrast provided subsequent modification of the intrinsic δ value through dehydration occurs. The apparent independence of contrast from the fixation method implies that other fixation methods could be explored moving forward, which may be more protective against excessive dehydration and crack formation in the tissue. EMA (ethanol, methanol and acetic acid), for example, shows greater preservation of tissue morphology than formalin, simultaneously fixing and dehydrating tissues^48^, all with fewer safety concerns than formalin. However, further investigation into such novel methods is needed.

## Conclusion

Tendon tissue was selected for this study due to its socioeconomic importance and because it presents challenges for traditional histological evaluation, motivating the exploration of new imaging approaches. XPCi was shown to offer a promising alternative, enabling the visualization of gross anatomy and tissue internal features, such as fascicles, in three dimensions. A simple, ethanol-based method has emerged as an effective and reproducible preparation protocol for laboratory based EI-XPCi imaging of tendon tissues. Overarchingly, laboratory based EI-XPCi CT has been demonstrated as a viable approach for the translational study of equine tendon tissues.

## Supplementary Information

Below is the link to the electronic supplementary material.


Supplementary Material 1


## Data Availability

The datasets generated during and/or analysed during the current study are available from the corresponding author on reasonable request. The 3D printer files (.stl format) along with detailed discussion of printer settings for the sample container (tube and lid) are freely available via the online repository Zenodo - https://doi.org/10.5281/zenodo.19354502.
