# Supplementary material for: Advancing three-dimensional tendon imaging using laboratory X-ray phase contrast techniques and refined sample preparation
Source: Sci Rep. 2026 Jun 13;16:27011. doi: 10.1038/s41598-026-57551-w (PMC13522437; doi:10.1038/s41598-026-57551-w)
Supplement: Supplementary file 1 [file 41598_2026_57551_MOESM1_ESM.docx]

Supplementary Information: Values used to calculate δ and β

Table 1 Chemical formula and density values used for estimating delta and beta values as displayed in Figure 8

| **Substance** | **Chemical Formula** | **Density (gcm^-3^)** |
| --- | --- | --- |
| Water | H_2_O | 1 |
| 100% EtOH | C_2_H_6_O | 0.79 |
| 70% EtOH | 70% C_2_H_6_O, 30% H_2_O | 0.88 |
| 10% NBF | 3.7% CH_2_0 96.3% H_2_O | 1.02 |

Table 2. Chemical formula, densities and weighting used to calculate delta and beta values of native fibrillar type 1 equine collagen.

| **Amino Acid** | **Proportion (/1000)** | **Density (g/cm3)** | **Formula** | | **δ** | | **β** | | **Weighted δ** | | **Weighted β** | |
| --- | --- | --- | --- | --- | --- | --- | --- | --- | --- | --- | --- | --- |
| Hydroxyproline | 104 | 1.45 | C_5_H_9_NO_3_ | | 9.93x10^-7^ | | 5.24x10^-10^ | | 1.03x10^-4^ | | 5.45 x10^-8^ | |
| Aspartic acid | 50 | 1.7 | C_4_H_7_NO_4_ | | 1.15 x10^-6^ | | 6.73 x10^-10^ | | 5.73x10^-5^ | | 3.37 x10^-8^ | |
| Threonine | 22 | 1.3 | C_4_H_9_NO_3_ | | 8.96 x10^-7^ | | 4.85 x10^-10^ | | 1.97x10^-5^ | | 1.07 x10^-8^ | |
| Serine | 39 | 1.6 | C_3_H_7_NO_3_ | | 1.09 x10^-6^ | | 6.26 x10^-10^ | | 4.26x10^-5^ | | 2.44 x10^-8^ | |
| Glutamine | 97 | 1.364 | C_5_H_10_N_2_O_2_ | | 9.41 x10^-7^ | | 4.62 x10^-10^ | | 9.13x10^-5^ | | 4.48 x10^-8^ | |
| Proline | 142 | 1.064 | C_5_H_9_NO_2_ | | 7.35 x10^-7^ | | 3.59 x10^-10^ | | 1.04 x10^-4^ | | 5.09 x10^-8^ | |
| Glycine | 219 | 1.161 | C_2_H_5_NO_2_ | | 7.93 x10^-7^ | | 4.51 x10^-10^ | | 1.74 x10^-4^ | | 9.88 x10^-8^ | |
| Alanine | 120 | 1.432 | C_3_H_7_NO_2_ | | 9.89 x10^-7^ | | 5.23 x10^-10^ | | 1.19 x10^-4^ | | 6.27 x10^-8^ | |
| Valine | 28 | 1.23 | C_5_H_11_NO_2_ | | 8.62 x10^-7^ | | 4.12 x10^-10^ | | 2.41 x10^-5^ | | 1.15 x10^-8^ | |
| Methionine | 4 | 1.178 | C_5_H_11_NO_2_S | | 8.11 x10^-7^ | | 1.51 x10^-9^ | | 3.24 x10^-6^ | | 6.04 x10^-9^ | |
| Isoleucine | 13 | 1.207 | C_6_H_13_NO_2_ | | 8.50 x10^-7^ | | 3.92 x10^-10^ | | 1.10 x10^-5^ | | 5.09 x10^-9^ | |
| Leucine | 32 | 1.293 | C_6_H_13_NO_2_ | | 9.10 x10^-7^ | | 4.19 x10^-10^ | | 2.91 x10^-5^ | | 1.34 x10^-8^ | |
| Tyrosine | 5 | 1.46 | C_6_H_11_NO_3_ | | 1.01 x10^-6^ | | 5.11 x10^-10^ | | 5.03 x10^-6^ | | 2.55 x10^-9^ | |
| Phenylalanine | 19 | 1.34 | C_9_H_11_NO_2_ | | 9.15 x10^-7^ | | 4.14 x10^-10^ | | 1.74 x10^-5^ | | 7.86 x10^-9^ | |
| Histidine | 11 | 1.49 | C_6_H_9_N_3_O_2_ | | 1.01 x10^-6^ | | 4.98 x10^-10^ | | 1.11 x10^-5^ | | 5.47 x10^-9^ | |
| Hydroxylysine | 13 | 1.268 | C_6_H_14_N_2_O_3_ | | 8.82 x10^-7^ | | 4.41 x10^-10^ | | 1.15 x10^-5^ | | 5.73 x10^-9^ | |
| Lysine | 26 | 1.13 | C_6_H_14_N_2_O_2_ | | 7.93 x10^-7^ | | 3.69 x10^-10^ | | 2.06 x10^-5^ | | 9.60 x10^-9^ | |
| Arginine | 56 | 1.42 | C_6_H_14_N_4_O_2_ | | 9.83 x10^-7^ | | 4.72 x10^-10^ | | 5.50 x10^-5^ | | 2.64 x10^-8^ | |
| Total | 1000 |  |  | |  | | **Weighted Averages** | | **8.99** **x10^-7^** | | **4.74** **x10^-10^** | |
